# 

**Published:** 1908-09

**Authors:** 


					CONTENTS.
Paee
Early History of the Bristol Royal Infirmary.
By J. Paul Bush, C.M.G.
193
^ Study of Ten Cases of Operation for Perforated Gastric Ulcer.
?oy Charles A. Morton, F.R.C.S.
207
^rgical Aid in Chronic Ulcer of the Stomach.
By J. A. Nixon, M.B. Cantab., M.R.C.P. Lond.
217
'he Aural Complications of Influenza and their Treatment.
By Harold F. Mole, F.R.C.S.
226
the Renal Changes in a Case of Hemorrhage into the Pons
with Consequent Hierh Blood Pressure.
By J. Michell Clarke, M.D., F.R.C.P.
230
^ Case of Severe Trigeminal Neuralgia Successfully Treated by
Excision of the Gasserian Ganglion.
By Ernest W. Hey Groves, M.S.Lond., F.R.C.S.
234
?me Complications of Ruptured Liver.
^ By Lionel Shingleton Smith, M.B.Cantab.
238
Pr,
?gress of the Medical Sciences
Medicine.
Carey Coombs, M.D. Lond. ...
241
ourgery.
1. Carwardine, M.rJ., M.b. L,ond., t.R.C.S.
246
Ophthalmology.
Cyril H. Walker, M.B., B.A.Cantab. ...
249
Reviews of Books
253
^itorial Notes
279
?tes on Preparations for the Sick
284
?*^e Library of the Bristol Medico-Chirurgical Society
1 "? 7 w,v- -
cal Medical Notes  r
2 '/'*

				

